# Supplementary material for: The potassium channel subunit KV1.8 (Kcna10) is essential for the distinctive outwardly rectifying conductances of type I and II vestibular hair cells
Source: bioRxiv. 2024 Jun 25:2023.11.21.563853. Originally published 2023 Nov 21. Preprint. [Version 2] doi: 10.1101/2023.11.21.563853 (PMC10690164; doi:10.1101/2023.11.21.563853)
Supplement: Supplementary file 1 [file NIHPP2023.11.21.563853v2-supplement-1.pdf]

bioRxiv preprint doi: https://doi.org/10.1101/2023.11.21.563853; this version posted June 25, 2024. The copyright holder for this preprint (which was not certified by peer review) is the author/funder, who has granted bioRxiv a license to display the preprint in perpetuity. It is made available under aCC-BY 4.0 International license.

Supplemental Figures

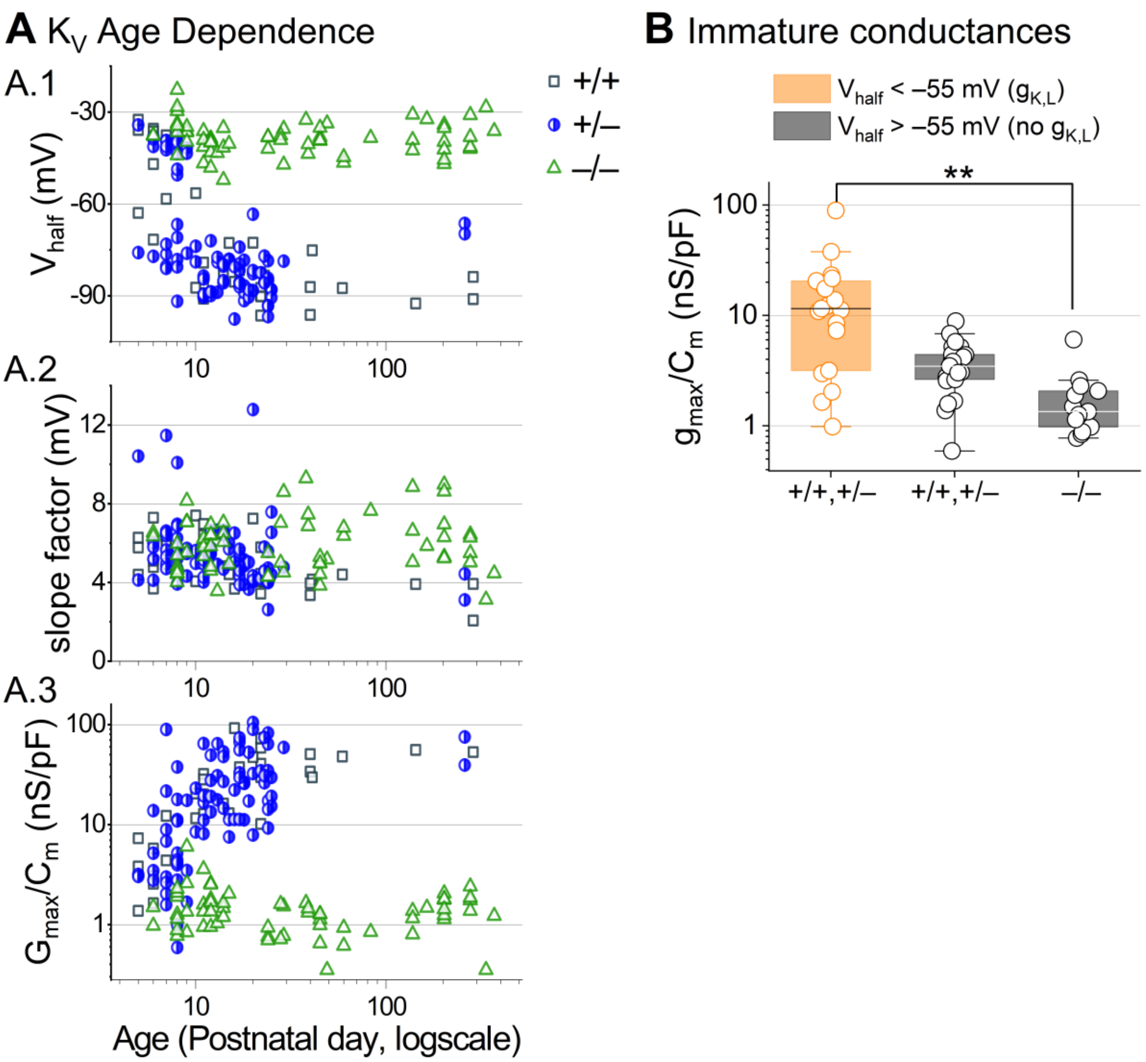

Supplemental Figure 1. Developmental changes in type I HC K_V conductances.
(**A**) Parameters from Boltzmann fits of tail G-V relations for type I HCs plotted against age.
(**B**) Conductance density is similar in young (P5-P10) type I HCs that lack $g_{K,L}$. $g_{K,L}$ is defined here as having a V_{half} negative to –55 mV. $K_V1.8^{+/+,+/–}$ *with* $g_{K,L}$, 17 ± 5 nS/pF (19); $K_V1.8^{+/+,+/–}$ *without* $g_{K,L}$, 3.7 ± 0.4 nS/pF (22); $K_V1.8^{–/–}$, 1.8 ± 0.4 nS/pF (13). $K_V1.8^{+/+,+/–}$ *with* $g_{K,L}$ vs. $K_V1.8^{–/–}$: p = 0.007, KWA, g 1.0.

bioRxiv preprint doi: https://doi.org/10.1101/2023.11.21.563853; this version posted June 25, 2024. The copyright holder for this preprint (which was not certified by peer review) is the author/funder, who has granted bioRxiv a license to display the preprint in perpetuity. It is made available under aCC-BY 4.0 International license.

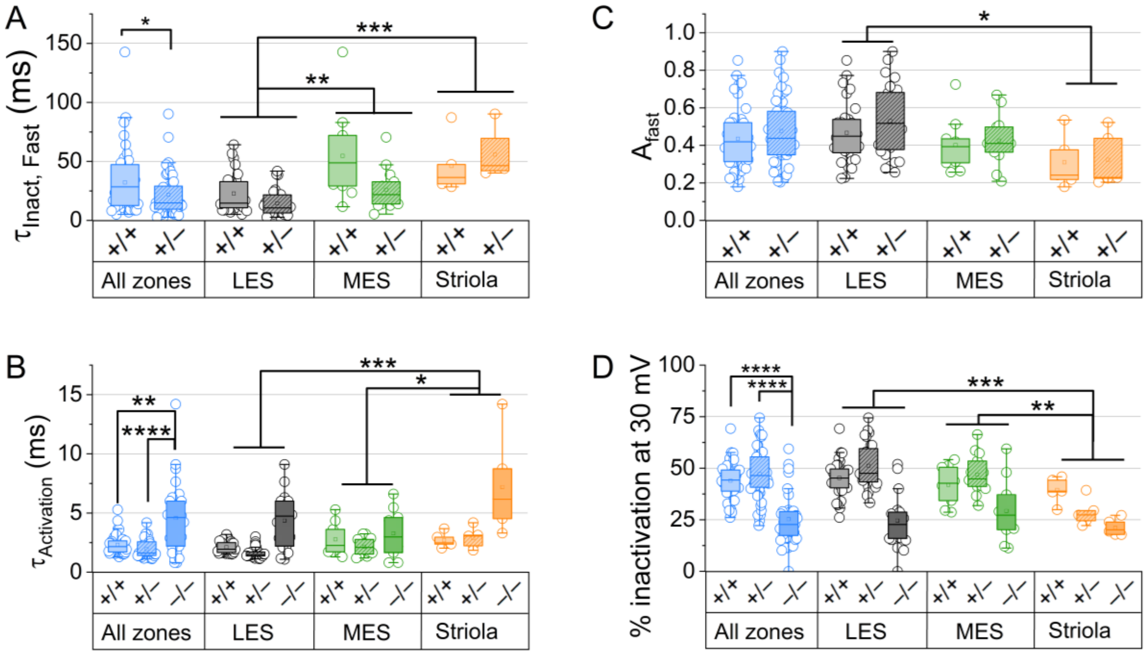

Supplemental Figure 2. **For type II HCs older than P12, K_V conductance activation and inactivation differed across zones and genotypes.**

(**A**) $\tau_{inact,Fast}$ at 30 mV was fastest in LES in $K_V1.8^{+/+}$ and $K_V1.8^{+/-}$ HCs, and faster in $K_V1.8^{+/-}$ than $K_V1.8^{+/+}$ HCs (see Table 3 for p-values).

(**B**) Fast inactivation was a larger fraction of the total in LES than striola.

(**C**) τ_{Act} at 30 mV was slower in $K_V1.8^{-/-}$ than $K_V1.8^{+/+}$ and $K_V1.8^{+/-}$, and slower in striola than LES and MES.

(**D**) Percent inactivation at 30 mV was lowest in striola (zone effect), and lowest in $K_V1.8^{-/-}$ HCs (genotype effect).

807

bioRxiv preprint doi: https://doi.org/10.1101/2023.11.21.563853; this version posted June 25, 2024. The copyright holder for this preprint (which was not certified by peer review) is the author/funder, who has granted bioRxiv a license to display the preprint in perpetuity. It is made available under aCC-BY 4.0 International license.

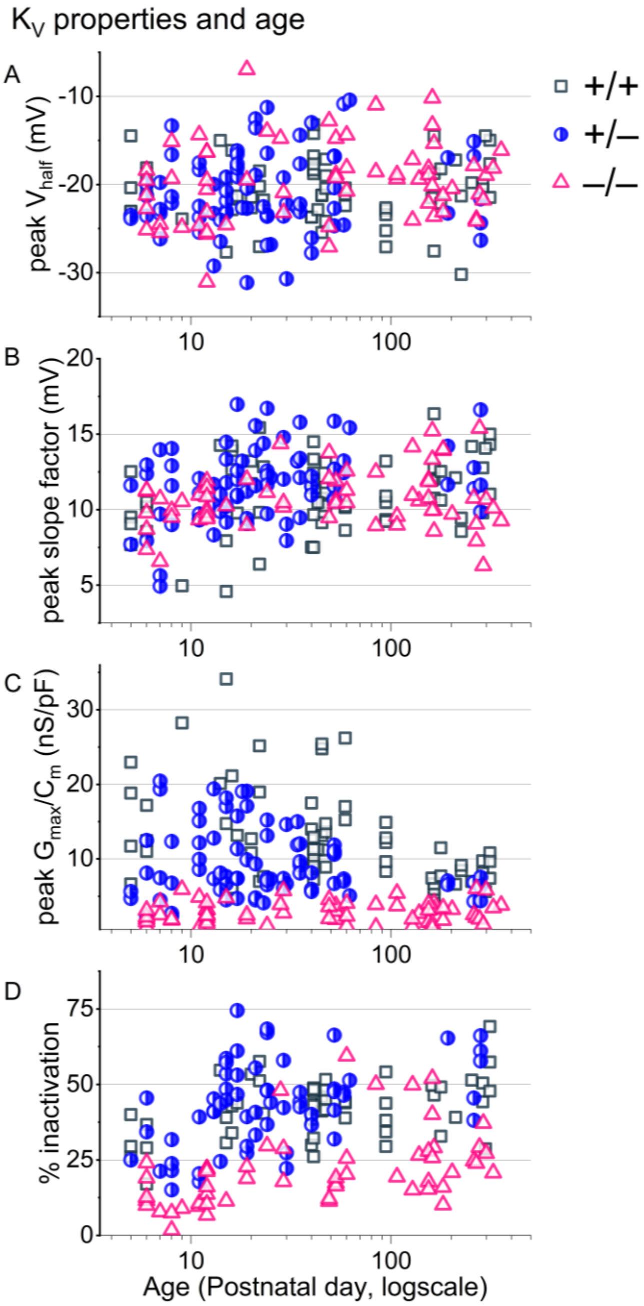

Supplemental Figure 3. For type II HCs older than P12, K_V conductances were stable. (**A-C**) Parameters from Boltzmann fits of peak G-V relations and (**D**) % inactivation at +30 mV plotted against age from all zones. Overlaid curves are smoothing cubic β-splines. Note the seven extrastriolar $K_V1.8^{-/-}$ type II HCs with % inactivation >30%.

bioRxiv preprint doi: https://doi.org/10.1101/2023.11.21.563853; this version posted June 25, 2024. The copyright holder for this preprint (which was not certified by peer review) is the author/funder, who has granted bioRxiv a license to display the preprint in perpetuity. It is made available under aCC-BY 4.0 International license.

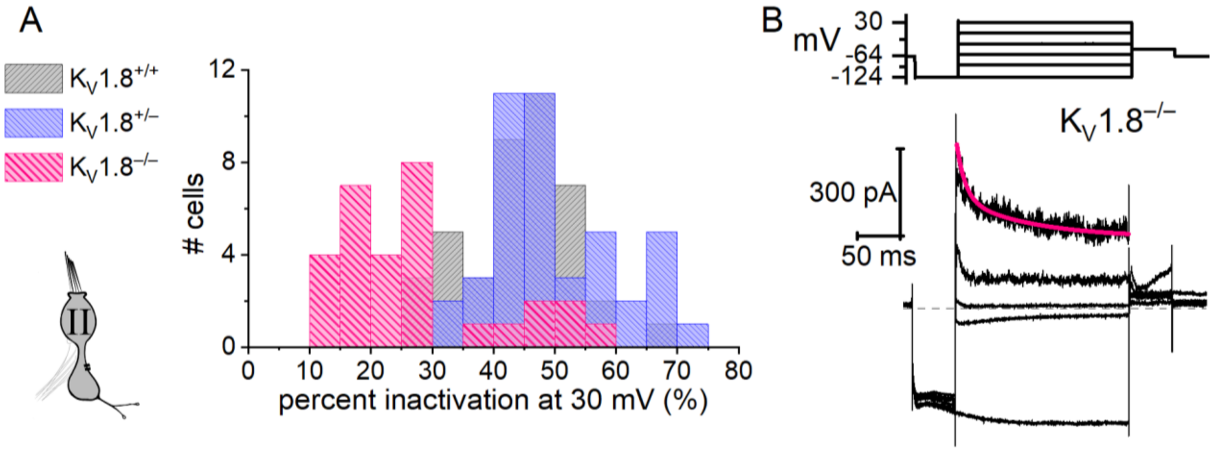

Supplemental Figure 4. A minority of extrastriolar $K_V1.8^{-/-}$ type II HCs had a very small fast-inactivating outward rectifier current.

(**A**) All extrastriolar $K_V1.8^{+/+,+/-}$ type II HCs inactivated by >30%. Most mature (>P12) extrastriolar $K_V1.8^{-/-}$ type II HCs inactivated by <30% but some inactivated by >30% (7/30, 23%) because they had fast inactivation (B).

(**B**) Exemplar residual fast inactivation ($\tau_{FastInact}$ = 10 ms at +30 mV). For the 7 cells in this group, $\tau_{FastInact}$ = 30 ± 6 ms, amplitude of fast inactivation = 310 ± 70 pA; activation peak V_{half} = –15 ± 2 mV and slope factor = 12.4 ± 0.9 mV. These parameters are similar to g_A but for the much smaller conductance (one-way ANOVAs).

bioRxiv preprint doi: https://doi.org/10.1101/2023.11.21.563853; this version posted June 25, 2024. The copyright holder for this preprint (which was not certified by peer review) is the author/funder, who has granted bioRxiv a license to display the preprint in perpetuity. It is made available under aCC-BY 4.0 International license.

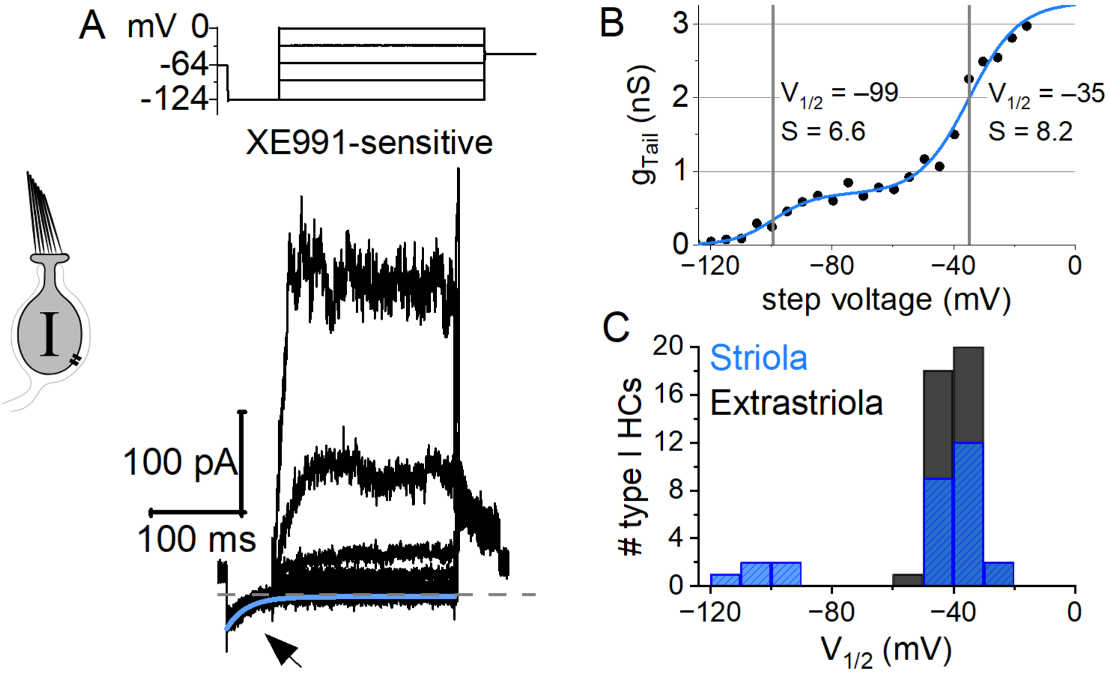

Supplemental Figure 5. A minority of striolar $K_V1.8^{-/-}$ type I HCs had a small low-voltage-activated outward rectifier current in addition to a more positively activating outward rectifier.

(**A**) Low-voltage-activated current from one cell was isolated by subtraction with 10 µM XE991 (P39), indicating that it was carried by K_V7 channels. Deactivation of XE991-sensitive current evoked by step from –64 mV to –124 mV *(arrow)* was fit with exponential decay (τ = 21 ms). (**B**) XE991-sensitive tail G-V curve of the XE991-blocked conductance (A) was fit with a sum of two Boltzmann equations: $G(V) = A_1/(1 + \exp((V_{half,1} - V)/S_1)) + A_2/(1 + \exp((V_{half,2} - V)/S_2))$.

(**C**) The low-voltage-activated $V_{half,1}$ component was only seen in striolar $K_V1.8^{-/-}$ type I HCs, and even there in the minority: 5/23; 22%; P6-P370). It was always seen together with a more positively activating outward rectifier. Boltzmann parameters, including (B): $A_1/(A_1+A_2)$ = 0.15 ± 0.04, $V_{half,1}$ = –106 ± 5 mV (n=5), S_1 = 3.8 ± 0.8 mV, $V_{half,2}$ = –41 ± 1 mV, S_2 = 7 ± 1 mV. Ages: P11, 39, 202, 202, 202. No extrastriolar type I HCs (0/45; P6-277) had a double activation tail G-V curve.

bioRxiv preprint doi: https://doi.org/10.1101/2023.11.21.563853; this version posted June 25, 2024. The copyright holder for this preprint (which was not certified by peer review) is the author/funder, who has granted bioRxiv a license to display the preprint in perpetuity. It is made available under aCC-BY 4.0 International license.

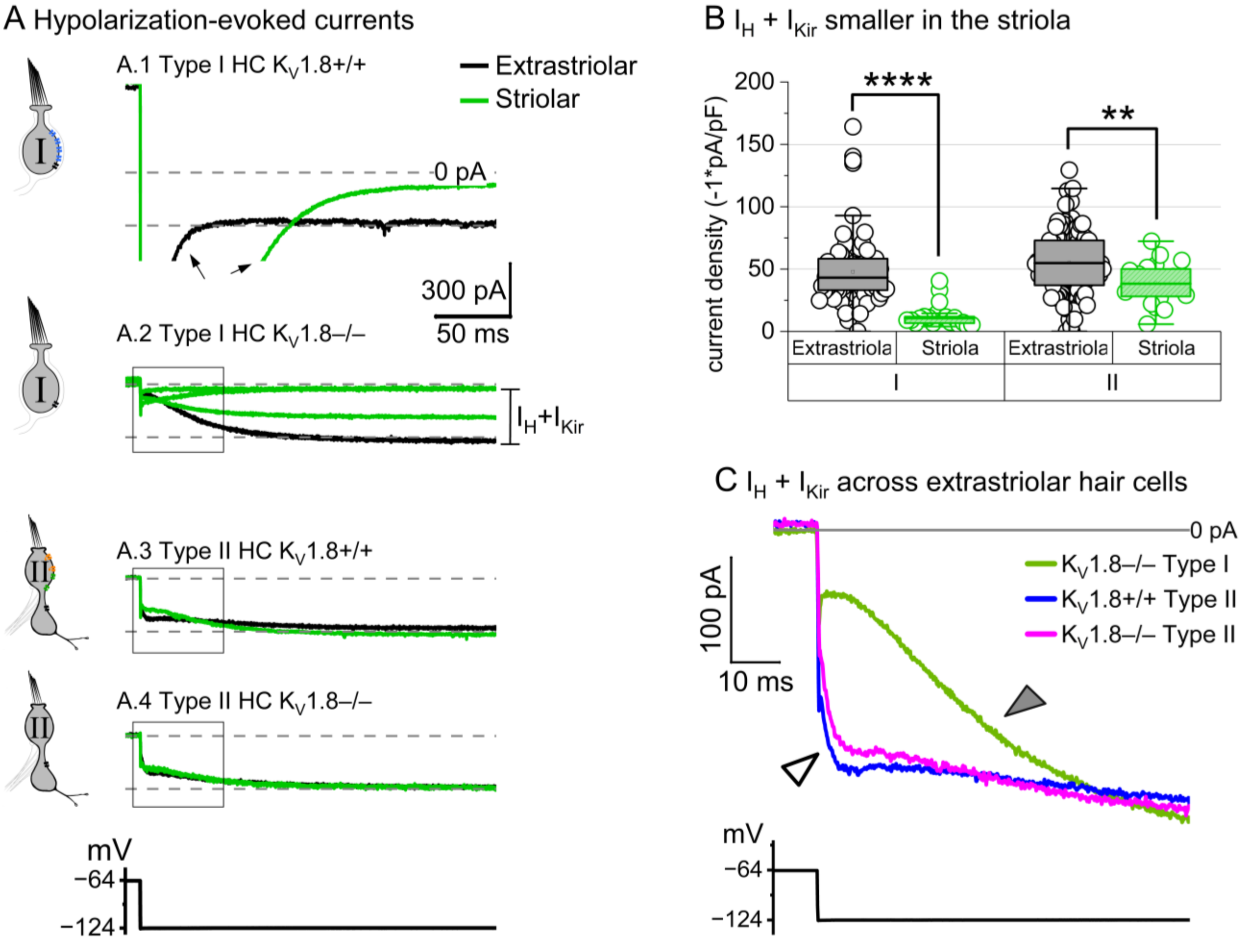

Supplemental Figure 6. No difference was detected in H (HCN) and Kir (fast inward rectifier) currents between $K_V1.8^{+/+}$ and $K_V1.8^{-/-}$ hair cells, consistent with a specific involvement of $K_V1.8$ in *Kcna10* expression.

(**A**) Hyperpolarizing voltage steps evoked I_{Kir} and I_{HCN} in $K_V1.8^{+/+,+/-,-/-}$ type I and II HCs. **A.1** *Arrows*, deactivation of $g_{K,L}$. **A.2-A.4** *Bracket,* the sum of I_H and I_{Kir} were measured as total inward current after 250 ms at –124 mV.

(**B**) Summed I_{KIR} and I_H density was the same across genotypes but smaller in striola than extrastriola (see Supplemental Table 3 for statistics).

(**C**) Inward currents seen at the onset of hyperpolarization, including I_{Kir}, were larger in type II HCs than type I. Magnification of boxed in inset from the extrastriolar cells in A.2-A.4. *Open arrowhead*, activation of fast inward rectifier, I_{Kir}; *filled arrowhead*, slower activation of I_{HCN}.

bioRxiv preprint doi: https://doi.org/10.1101/2023.11.21.563853; this version posted June 25, 2024. The copyright holder for this preprint (which was not certified by peer review) is the author/funder, who has granted bioRxiv a license to display the preprint in perpetuity. It is made available under aCC-BY 4.0 International license.

Supplemental Table 1. *Test of sex differences in hair cell K_V channel data.*

Cell Type	Parameter	Subgroup	Male vs Female posthoc p-value	Test
Type I HC	Tail V_{half}	+/+,+/– –/–	0.0023 ** [a] 0.57	Normal, homogeneous variance ANOVA: Genotype (2 levels), Sex (2 levels), Zone (2 levels), Genotype*Sex.
	Tail S	+/+,+/– –/–	0.98 0.58	ANOVA: Genotype (2 levels), Sex (2 levels), Zone (2 levels), Genotype*Sex. Normal, homogeneous variance
	Tail $g_{Density}$	+/+,+/– –/–	0.999 0.936	Normal, nonhomogeneous variance Welch ANOVA: Genotype*Sex
Type II HC	Peak V_{half}	+/+,+/– –/–	0.95 0.28	Normal, homogeneous variance ANOVA: Genotype (2 levels), Sex (2 levels), Zone (2 levels), Genotype*Sex.
	Peak S	+/+,+/– –/–	0.999 0.97	Normal, homogeneous variance ANOVA: Genotype (2 levels), Sex (2 levels), Zone (2 levels), Genotype*Sex.
	Peak $g_{Density}$	+/+,+/– –/–	0.64 0.43	Normal, nonhomogeneous variance Welch ANOVA: Genotype*Sex
	% inactivation at 30 mV	+/+,+/– –/–	0.98 0.82	Normal, homogeneous variance ANOVA: Genotype (2 levels), Sex (2 levels), Zone (2 levels), Genotype*Sex.

[a] g, 0.9. Male $K_V1.8^{+/+,+/-}$, –85 ± 1 mV (40) vs. Female $K_V1.8^{+/+,+/-}$, –79 ± 2 mV (12)

Supplemental Table 2. *Soma size of type I HCs. The perimeter of cross-sections of basolateral cell bodies was manually measured from immunohistochemistry sections with sufficient autofluorescence in the 488 channel to distinguish cell morphology, and anti-calretinin to label type II hair cells and calyx-only afferents. Cells were measured from two female littermates.*

Cell Type	Genotype	Age	Weight (g)	Perimeter (µm)	Test
Type I HC	+/+ –/–	P117 P117	24.9 25.2	47 ± 1 (19) 46 ± 1 (28)	ANOVA: p=0.1, power = 0.07

bioRxiv preprint doi: https://doi.org/10.1101/2023.11.21.563853; this version posted June 25, 2024. The copyright holder for this preprint (which was not certified by peer review) is the author/funder, who has granted bioRxiv a license to display the preprint in perpetuity. It is made available under aCC-BY 4.0 International license.

834

Supplemental Table 3. *Detected zonal but not genotype differences in hair cell I_{Kir} and I_H.*

Cell Type	Zone	I_H + I_{Kir} current density (-1*pA/pF)	$K_v1.8^{+/+,+/-}$ *vs* $K_v1.8^{-/-}$ p-value	ES *vs* Striola p-value	Test
Type I HC	ES<br>Striola	48 ± 3 (78)<br>13.0 ± 2 (19)	0.3	4E-9 **** [a]	Non-normal, KWA
Type II HC	ES<br>Striola	55 ± 2 (116)<br>39 ± 4 (20)	0.19 (0.25 power)	0.0058 ** [b]	Normal, homogeneous variance. 2-way ANOVA: Genotype (2 levels), Zone (2 levels)

[a] g 1.4
[b] g 0.6
