# Supplementary material for: The potassium channel subunit KV1.8 (Kcna10) is essential for the distinctive outwardly rectifying conductances of type I and II vestibular hair cells
Source: bioRxiv. 2023 Nov 21:2023.11.21.563853. Preprint. [Version 1] doi: 10.1101/2023.11.21.563853 (PMC10690164; doi:10.1101/2023.11.21.563853)
Supplement: Supplementary file 1 [file NIHPP2023.11.21.563853V1-supplement-1.pdf]

bioRxiv preprint doi: https://doi.org/10.1101/2023.11.21.563853; this version posted November 22, 2023. The copyright holder for this preprint (which was not certified by peer review) is the author/funder, who has granted bioRxiv a license to display the preprint in perpetuity. It is made available under aCC-BY 4.0 International license.

Supplemental Figures

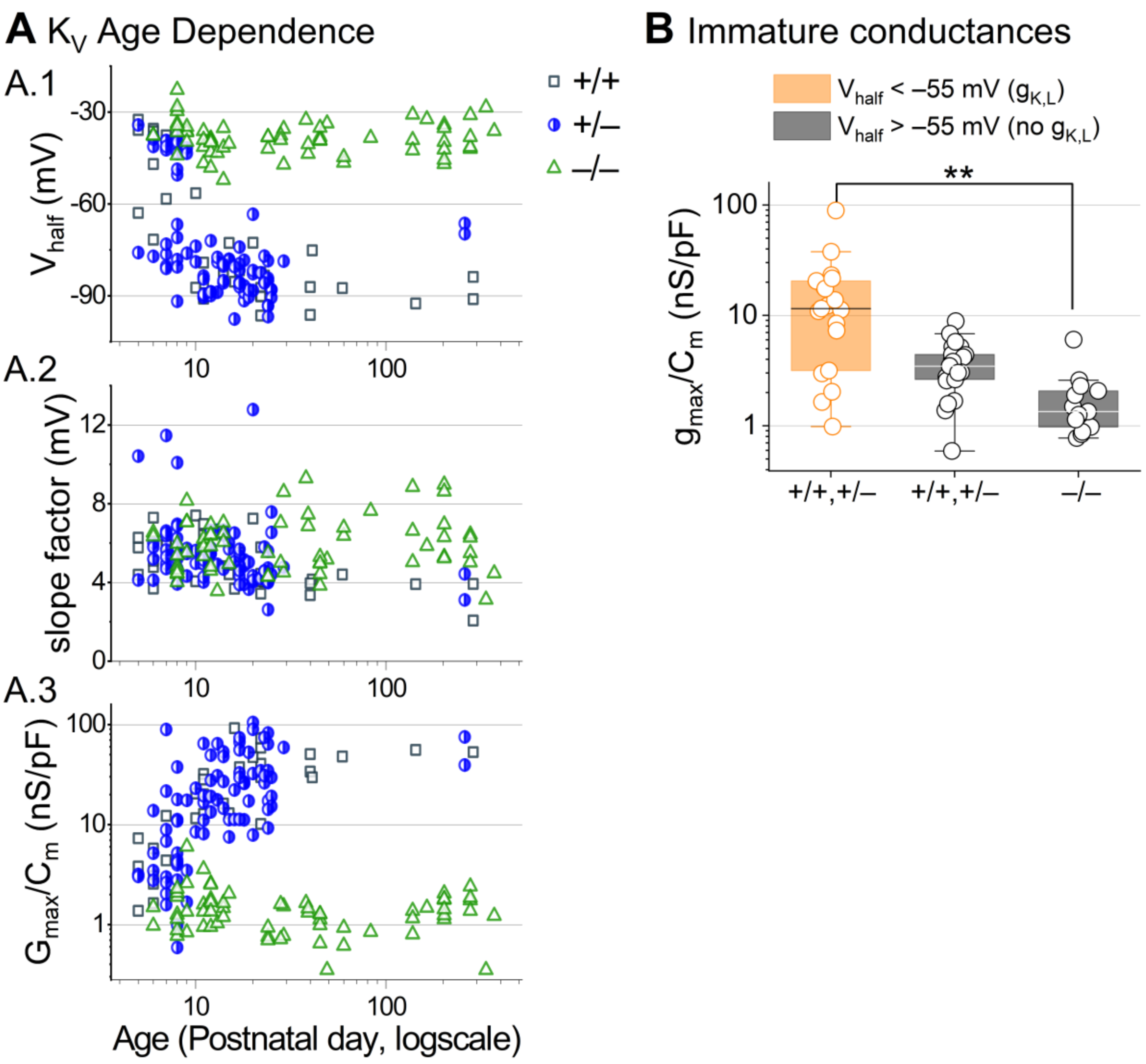

Supplemental Figure 1. Developmental changes in type I HC K_V conductances.
(**A**) Parameters from Boltzmann fits of tail G-V relations for type I HCs plotted against age.
(**B**) Conductance density is similar in young (P5-P10) type I HCs that lack $g_{K,L}$. $g_{K,L}$ is defined here as having a V_{half} negative to –55 mV. $K_V1.8^{+/+,+/-}$ *with* $g_{K,L}$, 17 ± 5 nS/pF (19); $K_V1.8^{+/+,+/-}$ *without* $g_{K,L}$, 3.7 ± 0.4 nS/pF (22); $K_V1.8^{-/-}$, 1.8 ± 0.4 nS/pF (13). $K_V1.8^{+/+,+/-}$ *with* $g_{K,L}$ vs. $K_V1.8^{-/-}$: p = 0.007, KWA, g 1.0.

bioRxiv preprint doi: https://doi.org/10.1101/2023.11.21.563853; this version posted November 22, 2023. The copyright holder for this preprint (which was not certified by peer review) is the author/funder, who has granted bioRxiv a license to display the preprint in perpetuity. It is made available under aCC-BY 4.0 International license.

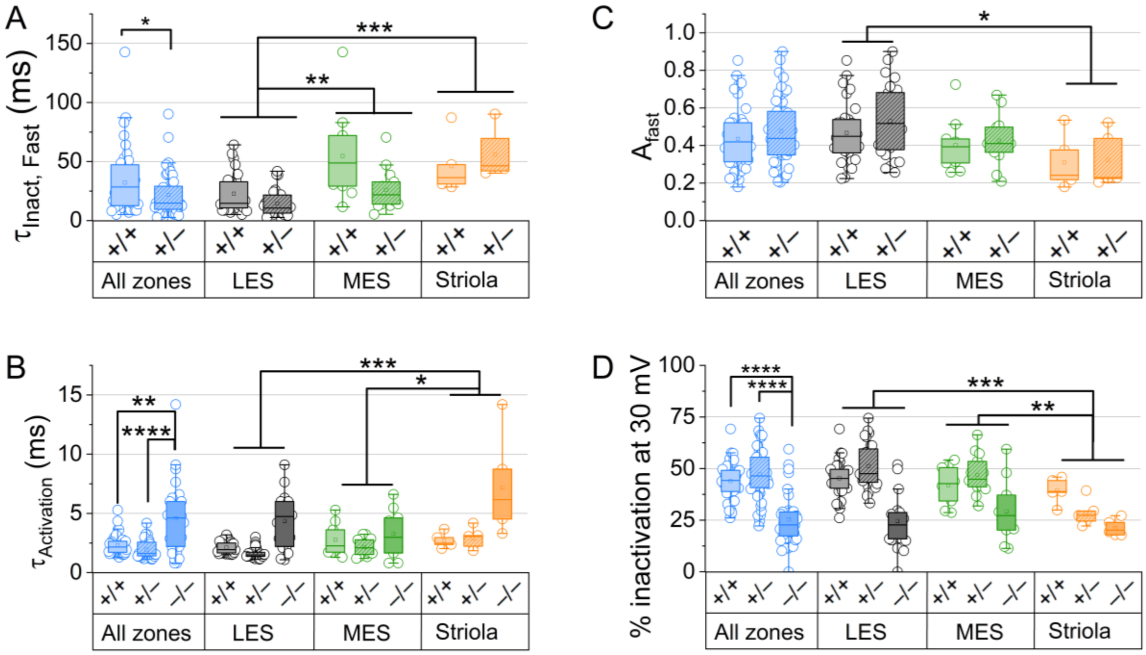

Supplemental Figure 2. **For type II HCs older than P12, K_V conductance activation and inactivation differed across zones and genotypes.**

(**A**) $\tau_{inact,Fast}$ at 30 mV was fastest in LES in $K_V1.8^{+/+}$ and $K_V1.8^{+/-}$ HCs, and faster in $K_V1.8^{+/-}$ than $K_V1.8^{+/+}$ HCs (see Table 3 for p-values).

(**B**) Fast inactivation was a larger fraction of the total in LES than striola.

(**C**) τ_{Act} at 30 mV was slower in $K_V1.8^{-/-}$ than $K_V1.8^{+/+}$ and $K_V1.8^{+/-}$, and slower in striola than LES and MES.

(**D**) Percent inactivation at 30 mV was lowest in striola (zone effect), and lowest in $K_V1.8^{-/-}$ HCs (genotype effect).

bioRxiv preprint doi: https://doi.org/10.1101/2023.11.21.563853; this version posted November 22, 2023. The copyright holder for this preprint (which was not certified by peer review) is the author/funder, who has granted bioRxiv a license to display the preprint in perpetuity. It is made available under aCC-BY 4.0 International license.

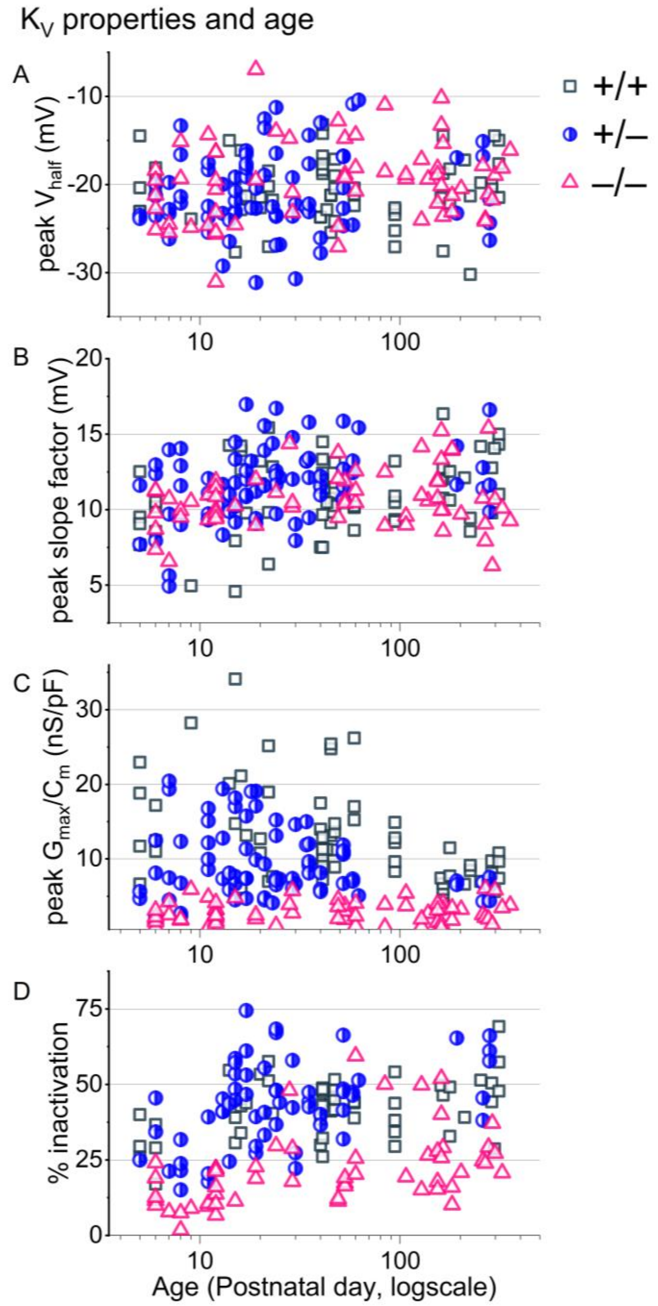

Supplemental Figure 3. For type II HCs older than P12, K_V conductances were stable. (**A-C**) Parameters from Boltzmann fits of peak G-V relations and (**D**) % inactivation at +30 mV plotted against age from all zones. Overlaid curves are smoothing cubic β-splines. Note the seven extrastriolar $K_V1.8^{-/-}$ type II HCs with % inactivation >30%.

bioRxiv preprint doi: https://doi.org/10.1101/2023.11.21.563853; this version posted November 22, 2023. The copyright holder for this preprint (which was not certified by peer review) is the author/funder, who has granted bioRxiv a license to display the preprint in perpetuity. It is made available under aCC-BY 4.0 International license.

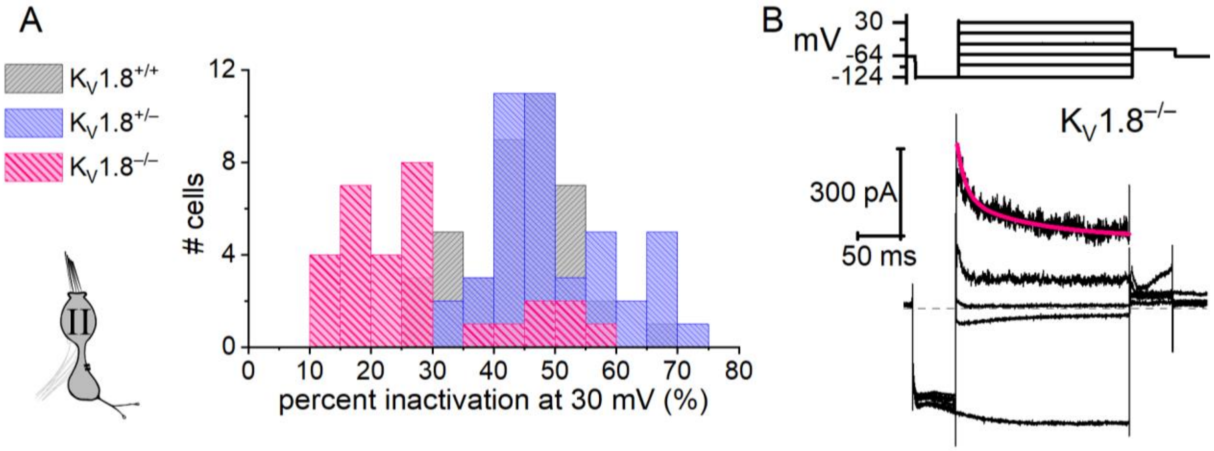

Supplemental Figure 4. A minority of extrastriolar $K_V1.8^{-/-}$ type II HCs had a very small fast-inactivating outward rectifier current.

(**A**) All extrastriolar $K_V1.8^{+/+,+/-}$ type II HCs inactivated by >30%. Most mature (>P12) extrastriolar $K_V1.8^{-/-}$ type II HCs inactivated by <30% but some inactivated by >30% (7/30, 23%) because they had fast inactivation (B).

(**B**) Exemplar residual fast inactivation ($\tau_{FastInact}$ = 10 ms at +30 mV). For the 7 cells in this group, $\tau_{FastInact}$ = 30 ± 6 ms, amplitude of fast inactivation = 310 ± 70 pA; activation peak V_{half} = –15 ± 2 mV and slope factor = 12.4 ± 0.9 mV. These parameters are similar to g_A but for the much smaller conductance (one-way ANOVAs).

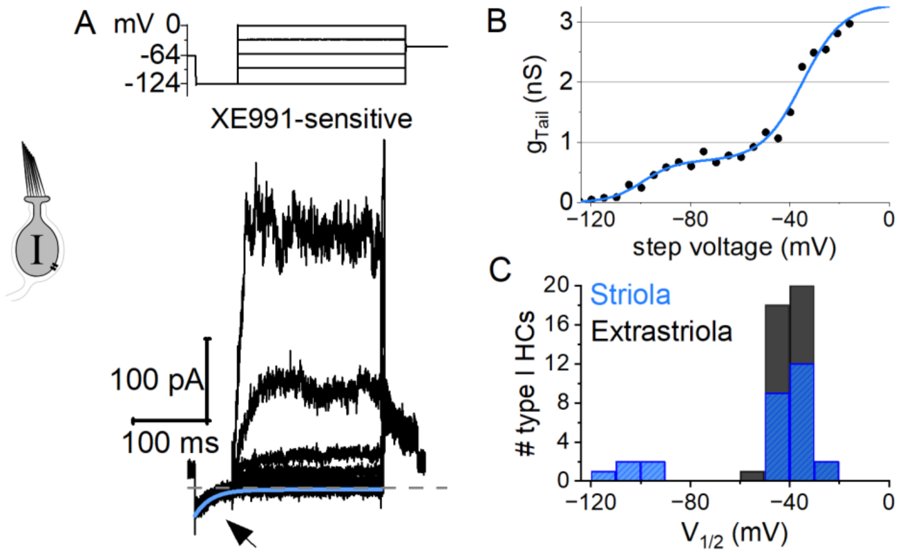

Supplemental Figure 5. A minority of striolar $K_V1.8^{-/-}$ type I HCs had a small low-voltage-activated outward rectifier current.

(**A**) Low-voltage-activated current from one cell was isolated by 10 µM XE991 (P39), suggesting it was a K_V7 current. Deactivation of XE991-sensitive current after step from –64 mV to –124 mV (*arrow*) was fit with exponential decay (τ = 21 ms).

(**B**) Tail G-V curve fit with a sum of two Boltzmann equations: $V_{half,1}$ = –102 ± 4 mV (n=5) and $V_{half,2}$ = –41 ± 1 mV. Ages: P11, 39, 202, 202, 202.

(**C**) Bimodal V_{half} distribution was specific to striolar type I HCs. 5/23 (22%; P6-P370) of striolar type I HCs had this low-voltage-activated component, but no extrastriolar type I HCs (0/45; P6-277).

bioRxiv preprint doi: https://doi.org/10.1101/2023.11.21.563853; this version posted November 22, 2023. The copyright holder for this preprint (which was not certified by peer review) is the author/funder, who has granted bioRxiv a license to display the preprint in perpetuity. It is made available under aCC-BY 4.0 International license.

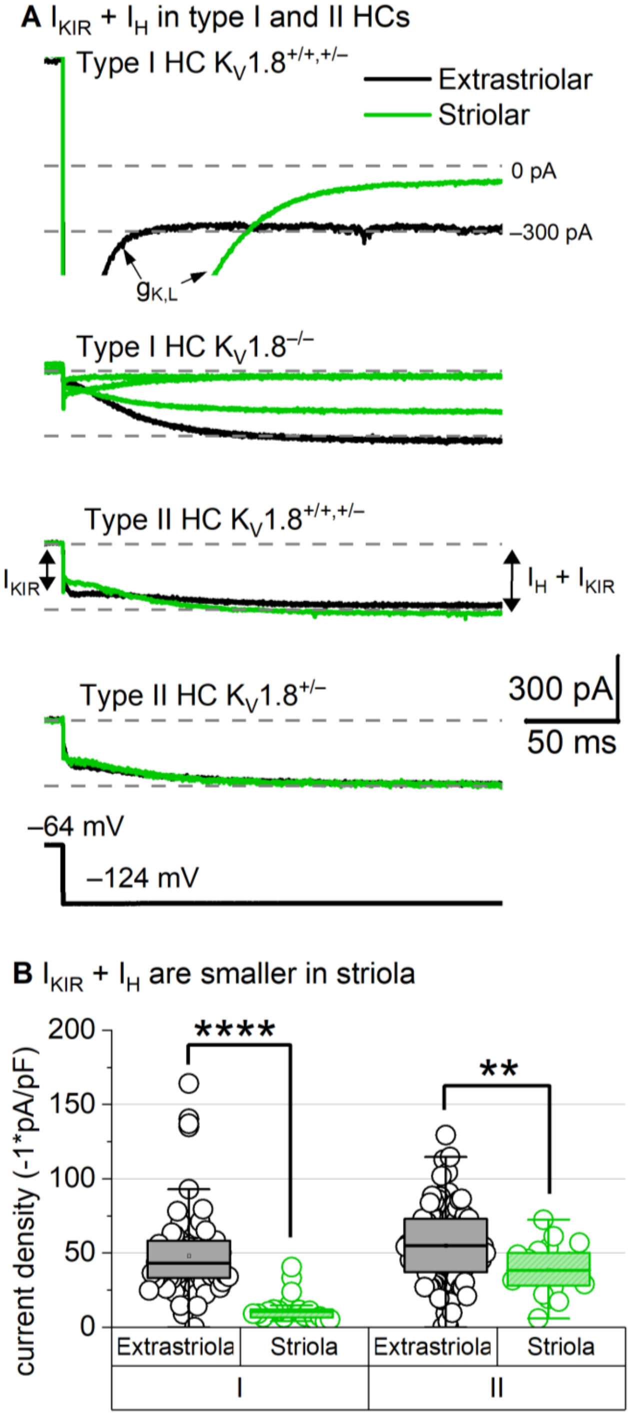

Supplemental Figure 6. No difference was detected in H (HCN) and KIR (fast inward rectifier) currents between $K_V1.8^{+/+}$ and $K_V1.8^{-/-}$ hair cells, consistent with a specific involvement of $K_V1.8$ in *Kcna10* expression.

(**A**) Hyperpolarizing voltage steps evoked I_{KIR} and I_{HCN} in $K_V1.8^{+/+,+/-,-/-}$ type I and II HCs. Note the prominent fast activation of I_{KIR} in type II but not type I HCs. Arrows in top panel show deactivation of $g_{K,L}$. I_H and I_{Kir} were measured as inward current after 250 ms at –124 mV.

(**B**) Summed I_{KIR} and I_H density was the same across genotypes but smaller in striola than extrastriola (see Supplemental Table 2 for statistics).

bioRxiv preprint doi: https://doi.org/10.1101/2023.11.21.563853; this version posted November 22, 2023. The copyright holder for this preprint (which was not certified by peer review) is the author/funder, who has granted bioRxiv a license to display the preprint in perpetuity. It is made available under aCC-BY 4.0 International license.

Supplemental Table 1. *Test of sex differences in hair cell K_V channel data.*

Cell Type	Parameter	Subgroup	Male vs Female posthoc p-value	Test
Type I HC	Tail V_{half}	+/+,+/–	0.0023 ** [a]	Normal, homogeneous variance<br>ANOVA: Genotype (2 levels), Sex (2 levels), Zone (2 levels), Genotype*Sex.
		–/–	0.57	
	Tail S	+/+,+/–	0.98	ANOVA: Genotype (2 levels), Sex (2 levels), Zone (2 levels), Genotype*Sex. Normal, homogeneous variance
		–/–	0.58	
	Tail $g_{Density}$	+/+,+/–	0.999	Normal, nonhomogeneous variance<br>Welch ANOVA: Genotype*Sex
		–/–	0.936	
Type II HC	Peak V_{half}	+/+,+/–	0.95	Normal, homogeneous variance<br>ANOVA: Genotype (2 levels), Sex (2 levels), Zone (2 levels), Genotype*Sex.
		–/–	0.28	
	Peak S	+/+,+/–	0.999	Normal, homogeneous variance<br>ANOVA: Genotype (2 levels), Sex (2 levels), Zone (2 levels), Genotype*Sex.
		–/–	0.97	
	Peak $g_{Density}$	+/+,+/–	0.64	Normal, nonhomogeneous variance<br>Welch ANOVA: Genotype*Sex
		–/–	0.43	
	% inactivation at 30 mV	+/+,+/–	0.98	Normal, homogeneous variance<br>ANOVA: Genotype (2 levels), Sex (2 levels), Zone (2 levels), Genotype*Sex.
		–/–	0.82	

[a] g, 0.9. Male $K_V1.8^{+/+,+/-}$, –85 ± 1 mV (40) vs. Female $K_V1.8^{+/+,+/-}$, –79 ± 2 mV (12)

Supplemental Table 2. *Detected zonal but not genotype differences in hair cell I_{KIR} and I_H.*

Cell Type	Zone	$I_H + I_{Kir}$ current density (-1*pA/pF)	$K_V1.8^{+/+,+/-}$ *vs* $K_V1.8^{-/-}$ p-value	ES *vs* Striola p-value	Test
Type I HC	ES<br>Striola	48 ± 3 (78)<br>13.0 ± 2 (19)	0.3	4E-9 **** [a]	Non-normal, KWA
Type II HC	ES<br>Striola	55 ± 2 (116)<br>39 ± 4 (20)	0.19 (0.25 power)	0.0058 ** [b]	Normal, homogeneous variance. 2-way ANOVA: Genotype (2 levels), Zone (2 levels)

[a] g 1.4
[b] g 0.6
